# Physicochemical, Sensorial and Calcium Bioavailability of Jelly Prepared Using Fish Gelatin in Combination with Furcellaran and Calcium L-Threonate

**DOI:** 10.3390/gels12010026

**Published:** 2025-12-28

**Authors:** Tanyamon Petcharat, Manat Chaijan, Sylvia Indriani, Supatra Karnjanapratum, Nilesh Nirmal, Jaspreet Singh, Ihlana Nairfana, Sitthipong Nalinanon

**Affiliations:** 1 School of Food Industry, King Mongkut’s Institute of Technology Ladkrabang, Ladkrabang, Bangkok 10520, Thailand; tanyamon.pe@wu.ac.th; 2 Professional Culinary Arts Program, School of Management, Walailak University, Thasala, Nakhon Si Thammarat 80161, Thailand; 3 Food Technology and Innovation Research Centre of Excellence, School of Agricultural Technology and Food Industry, Walailak University, Thasala, Nakhon Si Thammarat 80161, Thailand; cmanat@wu.ac.th; 4 School of Animal Technology and Innovation, Institute of Agricultural Technology, Suranaree University of Technology, Nakhon Ratchasima 30000, Thailand; indrianisylvia@gmail.com; 5 Division of Marine Product Technology, Faculty of Agro-Industry, Chiang Mai University, Chiang Mai 50100, Thailand; supatra.ka@cmu.ac.th; 6 Institute of Nutrition, Mahidol University, 999 Phutthamonthon 4 Road, Salaya, Nakhon Pathom 73170, Thailand; nilesh.nir@mahidol.ac.th; 7 School of Food and Advanced Technology, Massey University, Palmerston North 4442, New Zealand; j.x.singh@massey.ac.nz (J.S.); i.nairfana@massey.ac.nz (I.N.)

**Keywords:** furcellaran, fish gelatin, calcium fortification, gelling property, calcium-fortified jelly

## Abstract

Confectionery products, specifically jelly and gummy, require optimized structural, thermal, and nutritional properties for functionality and consumer acceptance. This study investigated the impact of furcellaran (FUR) and calcium L-threonate (Ca) on the physicochemical and the sensory properties of fish gelatin-based jelly (JFG). Furcellaran modestly enhanced gel strength and hardness, while its combination with calcium L-threonate produced synergistic improvements, with JFG-FUR-Ca achieving the highest gel strength (947.63 g) and hardness (78.14 N). Microstructural and intermolecular force analyses indicated that Ca^2+^ bridging between gelatin and furcellaran promoted ionic and hydrogen bonding, forming a dense and thermostable network. The combined incorporation of furcellaran and calcium L-threonate enhanced the rheological properties while preserving low syneresis. Sensory evaluation revealed minor reductions; however, overall acceptability was higher than 7. Calcium bioavailability after digestion through the gastrointestinal tract model remained high (70–80%), confirming effective calcium fortification. The synergistic incorporation of furcellaran and calcium L-threonate effectively improved the structural integrity, thermal stability, and calcium bioavailability of fish gelatin-based jelly, while maintaining acceptable sensory qualities, highlighting its potential as a functional calcium-fortified confectionery product.

## 1. Introduction

Confectionery products are enjoyed by all age groups, specifically jelly and gummy varieties, which are especially attractive to younger consumers owing to their sweetness, chewy texture, and visually appealing presentation [1,2]. Jelly is particularly favored for its delightful taste, smooth gel texture, and ease of consumption. The manufacturing process typically entails thermal treatment, followed by filling, shaping, and cooling phases to achieve the final gel product [3].

Gelatin is widely utilized in dairy products, bakery items, and confectionery, especially in jelly products, because it contributes to desirable flavor, enhances product stability and improves texture characteristics such as chewiness, while also offering nutritional benefits [4]. As a natural polypeptide derived from the partial hydrolysis of collagen obtained from mammalian or fish sources, gelatin is also free of fat and cholesterol [5]. Recently, concerns have arisen regarding the use of gelatin from terrestrial animals due to potential risks of disease transmission, including bovine spongiform encephalopathy and foot-and-mouth disease. Moreover, porcine gelatin is not acceptable to particular religious communities. These issues have encouraged the exploration of fish gelatin as an alternative source. Significantly, gelatin could be recovered from fish-processing residues, helping to reduce waste and environmental impact [6,7]. However, a key limitation of fish gelatin is its comparatively low gel strength; it cannot be set at room temperature (25 °C) and lowered in thermostability [8]. These primarily result from its lower content of proline and hydroxyproline [9,10], which reduces the formation of hydrogen bonds necessary to stabilize the gelatin triple-helix structure and maintain an ordered network, thereby limiting its practical applications. Among the various sustainable and cost-effective strategies to address the limitations of fish gelatin, the incorporation of several ionic hydrocolloids has emerged as a promising approach to enhance its gelation properties. Carrageenan [10,11,12], hyaluronic acid [13], pectin [14,15], sodium alginate [16], gellan [7,8,17], and chitosan [18] have been used for improving the structure of fish gelatin hydrogels. Most efforts to improve fish gelatin-based hydrogel products have concentrated on enhancing the textural attributes—particularly gel strength and thermal stability—through the incorporation of hydrocolloids. Some studies have also aimed to reduce sugar content to meet the demands of health-conscious consumers [1]. Despite these efforts, the development of jelly products that simultaneously optimize both textural properties and nutritional value remains limited.

Furcellaran is a sulfated anionic hydrocolloid extracted from red algae (*Furcellaria lumbricalis*). It is structurally and functionally similar to κ-carrageenan but differs in the quantity of sulfate ester groups [19]. This anionic hydrocolloid exhibits excellent water solubility, forms gels rapidly, and is biodegradable, biocompatible, and non-toxic [20]. Previous research has demonstrated that incorporating furcellaran into fish gelatin could successfully improve the hydrogel thermal stability and textural quality [21]. The addition of calcium ions exhibited a significant synergistic effect via enhancing the gelling characteristics of furcellaran [22]. Calcium chloride (CaCl_2_) is one of the most widely utilized calcium salts for improving the gel characteristics of gelatin, particularly when combined with hydrocolloids [23]. Nevertheless, the excessive addition of CaCl_2_ can impart undesirable bitter or salty flavors, thereby reducing consumer acceptability [24]. Recently, calcium L-threonate has gained attention as a potential alternative calcium source. This compound is a white, non-hygroscopic powder that lacks any pronounced odor or taste [25,26]. Apart from its favorable sensory attributes, calcium L-threonate offers a highly bioavailable form of calcium, which plays a vital role in preventing osteoporosis and other disorders associated with calcium deficiency [27].

The incorporation of furcellaran with fish gelatin represents a promising strategy to enhance the structural and thermal stability of gelatin-based hydrogels. At the same time, calcium L-threonate serves as a crosslinker and calcium source for nutritional fortification. Nevertheless, the interaction between calcium salts, hydrocolloids, and gelatin matrices under the acidic conditions typical of jelly systems remains a challenge that warrants further investigation. This approach presents an innovative opportunity to bridge the gap between indulgent confectionery and functional, health-promoting foods. Therefore, the present study was undertaken to investigate the physicochemical, sensorial properties, and calcium bioavailability of jelly prepared using fish gelatin in combination with furcellaran and fortified with calcium L-threonate.

## 2. Results and Discussion

### 2.1. Textural Properties of Formulated Jellies

#### 2.1.1. Gel Strength

**Figure 1:**
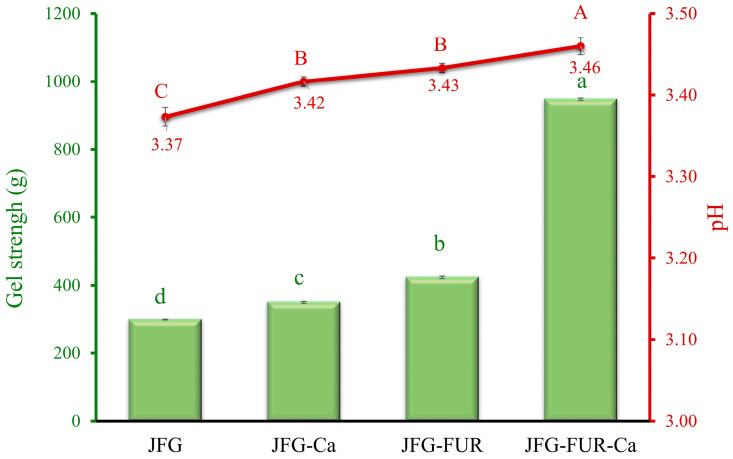
Gel strength and pH of jelly samples prepared using fish gelatin with and without other additives (furcellaran and calcium L-threonate). Bars and the line represent gel strength and pH, respectively. The bars and line are presented as the standard deviation (*n* = 10). Lowercase or uppercase letters on the bar and line indicate significant differences (*p* < 0.05). JFG = fish gelatin jelly, JFG-Ca = fish gelatin jelly with calcium L-threonate, JFG-FUR = fish gelatin jelly containing furcellaran, and JFG-FUR-Ca = fish gelatin–furcellaran jelly incorporated with calcium L-threonate.

Gel strength is a key attribute used to evaluate gelatin quality and its suitability for various applications. Notably, insufficient gel strength could limit the functional uses of gelatin-based gels [28]. For jelly-type confectionery products, moderate gel strength is desirable to ensure shape retention while maintaining softness and melt-in-mouth characteristics, whereas gummy products typically require much higher rigidity and chewiness. The gel strength of various jellies prepared using fish gelatin with or without other additives is shown in Figure 1. The JFG (fish gelatin jelly without incorporation) exhibited the lowest gel strength (298.71 g) (*p* < 0.05), while the addition of calcium L-threonate (JFG-Ca) marginally increased the gel strength up to 349.46 g. The pH values of the resulting jelly samples ranged from 3.37 to 3.46 (as shown in Figure 1), which are well below the isoelectric point of fish gelatin (pI ≈ 8.8) [29]. Under these acidic conditions, fish gelatin molecules predominantly carried positive charges. Excessively charged leads to high electrostatic repulsion among chains. This repulsion might cause chain expansion and hinder their ability to align and form helical structures or a stable three-dimensional network [30]. Consequently, the JFG sample exhibited the weakest gel structure among all formulations.

The incorporation of calcium L-threonate slightly increased the pH of the jelly, which might help reduce electrostatic repulsion among gelatin chains, thereby contributing to the improved gel strength observed in JFG-Ca. Moreover, incorporating furcellaran into the fish gelatin jelly (JFG-FUR) further improved its gelling properties. The highest gel strength was obtained in the jelly containing both furcellaran and calcium L-threonate (JFG-FUR-Ca) (*p* < 0.05). This improvement is likely attributable to interactions between the positively charged fish gelatin molecules and the negatively charged sulfate groups present in furcellaran, which help strengthen the gel matrix under acidic conditions [21]. The presence of calcium further amplified this effect, presumably by forming an ionic bond between furcellaran sulfate groups [31] and gelatin peptide chains, thereby reinforcing the gel network and promoting greater three-dimensional stability. This synergistic enhancement is likely due to increased cross-linking interactions between furcellaran, gelatin, and calcium ions, producing a more compact and stable network structure. Similar improvements in protein-polysaccharide matrix strength upon ionic addition have been reported in previous studies [7,29,32]. These reports supported the role of calcium in helping form intermolecular links in mixed hydrogel systems. Therefore, the combined addition of furcellaran and calcium L-threonate could provide a synergistic effect, resulting in markedly improved textural characteristics of the jelly products.

#### 2.1.2. Texture Profile Analysis (TPA)

Textural attributes are critical indicators of the sensory and functional qualities associated with the mechanical and structural properties of foods. Instrumental texture analysis enables the simulation of structural changes that occur during mastication, thereby providing insight into the in-mouth behavior of colloidal systems [2,33,34]. The textural profile analysis (TPA) of the jelly samples is summarized in Table 1. Significant differences (*p* < 0.05) were observed for all evaluated parameters. The JFG sample showed the lowest hardness value (26.21 N) (*p* < 0.05), indicating a weak gel network. When calcium L-threonate was added (JFG-Ca), hardness increased slightly to 30.55 N. A marked increase in hardness was observed in the JFG-FUR sample. The result suggested that furcellaran contributed strongly to network formation. The highest hardness was obtained in the JFG-FUR-Ca sample (78.14 N) (*p* < 0.05). This enhancement might be explained by interactions between positively charged fish gelatin and negatively charged sulfate groups in furcellaran [21]. In addition, calcium ions could act as salt-bridging agents, promoting additional cross-linking between biopolymers and forming a more compact gel structure [35]. It was noted that the highest hardness observed in gels containing both furcellaran and calcium L-threonate corresponded with the highest gel strength shown in Figure 1. This trend reconfirmed that the gel matrix became more reinforced. Springiness refers to the ability of a gel to regain its original shape after deformation [36], while cohesiveness reflects the internal bonding strength that enables the material to withstand subsequent compression cycles [37]. Incorporation of calcium into the jelly (JFG-Ca) did not result in significant changes in either springiness or cohesiveness compared with the control (JFG), indicating that calcium alone had minimal impact on the elastic recovery and internal structural integrity of the gel. In contrast, the addition of furcellaran, either alone or in combination with calcium L-threonate, led to a marked decrease in both springiness and cohesiveness (*p* < 0.05). This reduction suggests that furcellaran incorporation may alter network packing or disrupt elastic recovery, thereby diminishing the gel’s ability to maintain cohesion under mechanical stress. Although the incorporation of furcellaran with calcium ions produced a firmer gel, its ability to recover after deformation decreased. This reduced elastic response might be related to the formation of a denser and less flexible network, especially at higher calcium levels. A similar tendency was reported by Huang, Tu, Sha, Wang, Hu and Hu [32], who found that gels formed with fish gelatin and low-methoxyl pectin showed decreased springiness as CaCl_2_ concentration increased.

Moreover, gumminess and chewiness showed similar trends to hardness. JFG showed the lowest gumminess and chewiness (*p* < 0.05). These values increased slightly after calcium addition (JFG-Ca) and more substantially with furcellaran incorporation (JFG-FUR). The highest gumminess and chewiness were observed in JFG-FUR-Ca, indicating that this sample required greater force to deform and break down during mastication [38]. The elevation in both parameters (furcellaran and calcium L-threonate) is consistent with the formation of a more compact cross-linked network. In summary, the findings demonstrated that furcellaran substantially enhanced the textural characteristics of fish-gelatin-based jelly, particularly hardness, gumminess, and chewiness. The presence of calcium further reinforces the gel more likely through ionic bridging between sulfate groups of furcellaran and gelatin peptides, thereby stabilizing the three-dimensional hydrogel network. Although springiness and cohesiveness decreased slightly, the combined addition of furcellaran and calcium L-threonate yielded the most desirable texture attributes overall.

### 2.2. Intermolecular Force of Jelly Samples

**Figure 2:**
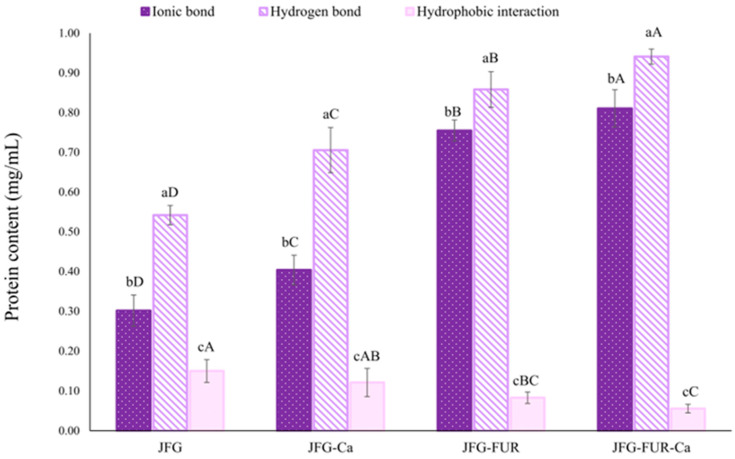
Intermolecular interaction forces of jelly samples prepared using fish gelatin with and without other additives (furcellaran and calcium L-threonate). Bars represent the standard deviation (*n* = 3). Lowercase letters on the bars indicate significant differences (*p* < 0.05) among different types of interaction within the same sample (intragroup comparison), while uppercase letters indicate significant differences (*p* < 0.05) of the same interaction type across different samples (intergroup comparison). JFG = fish gelatin jelly, JFG-Ca = fish gelatin jelly with calcium L-threonate, JFG-FUR = fish gelatin jelly containing furcellaran, and JFG-FUR-Ca = fish gelatin–furcellaran jelly incorporated with calcium L-threonate.

Fractional dissolution analysis serves as an indirect method used to evaluate protein solubility in the supernatant. The degree of solubility reflects the extent of protein aggregation and provides insight into molecular interactions—such as electrostatic attraction, hydrophobic forces, and disulfide linkages—that contribute to gel stability [39]. Higher protein solubility is associated with stronger intermolecular interactions and more robust molecular associations [40]. Therefore, the contribution of individual intermolecular forces inferred from this method represents a relative tendency rather than an absolute quantification. Figure 2 illustrates the relative contributions of intermolecular forces, including ionic bonds, hydrogen bonds, and hydrophobic interactions within different jelly samples. A notable difference in intermolecular interactions was observed among the jelly samples. In all formulations, ionic bonding and hydrogen bonding constituted the principal forces stabilizing the gel network, whereas hydrophobic interactions contributed to a much lesser extent (*p* < 0.05). However, it should be noted that these interaction types are mutually interrelated, and changes in one type of interaction might influence the others. Incorporation of calcium L-threonate (JFG-Ca) significantly increased protein solubility associated with ionic and hydrogen bond formation compared with JFG (*p* < 0.05). This enhancement might be attributed to the ability of calcium ions to act as bridging agents, forming ionic bonds between negatively charged sites on furcellaran or gelatin chains, thereby strengthening the network structure. A greater increase in ionic and hydrogen bonding–associated solubility was observed upon the addition of furcellaran alone (JFG-FUR). This behavior might be explained by the presence of highly anionic sulfate groups in furcellaran, which strongly interact with the positively charged domains of fish gelatin under acidic conditions. Such interactions facilitate closer chain packing and strengthen polymer–polymer association. In addition, both intramolecular and intermolecular hydrogen bonds might be established during gelation between functional groups of fish gelatin (e.g., hydroxyl, carboxyl, and amine groups, along with certain counterions) and those of furcellaran (e.g., sulfate and hydroxyl groups). Moreover, the increased solubility observed in the presence of urea solution might therefore arise not only from disruption of hydrogen bonds, but also from the destabilization of ordered protein–polysaccharide assemblies or partial unfolding of gelatin chains. This phenomenon was supported by the observed increase in protein solubility, indicating intensified molecular interactions [39,41,42].

The most pronounced effects were found in the formulation containing both furcellaran and calcium L-threonate. The JFG-FUR-Ca jelly sample exhibited the highest levels of ionic and hydrogen bonding (*p* < 0.05). This synergistic response reconfirmed that calcium ions further enhance electrostatic attraction by forming ionic bridges between sulfate groups on furcellaran and amino groups on gelatin chains [43]. These additional cross-links contribute to an increasingly compact and rigid three-dimensional network structure. Sow, Peh, Pekerti, Fu, Bansal and Yang [43] demonstrated that the incorporation of calcium ions (supplied by calcium chloride) into a gellan–gelatin system modified the balance between attractive and repulsive interactions. This perturbation promoted structural rearrangement at the nanoscale, thereby facilitating the establishment of hydrogen bonds between the amino groups of gelatins and the carboxyl and hydroxyl functionalities of gellan gum. According to the findings, the highest ionic and hydrogen bonds were coincidental with the highest gel strength (Figure 1) and hardness (Table 1) observed in JFG-FUR-Ca. This association reinforces the notion that enhanced intermolecular bonding played a key role in improving textural characteristics. Hydrophobic interaction was the least dominant force in all samples, and its contribution decreased slightly after furcellaran incorporation. This reduction could indicate that stronger ionic and hydrogen bonding limited the need for hydrophobic association in maintaining network integrity. Since hydrogen and ionic bonds provided more effective stabilization, hydrophobic forces played only a supporting role in the system. Overall, these results suggested that ionic and hydrogen bonds are the predominant contributors of the gel structure in furcellaran–fish gelatin systems, while hydrophobic interactions provided secondary support. The combination of furcellaran with calcium L-threonate greatly reinforced intermolecular associations, leading to a stronger gel network.

### 2.3. Syneresis of Jelly Samples

Syneresis refers to the release of unbound water from a gel matrix, occurring as a normal post-gelation process. However, this behavior is typically undesirable in food gels. Syneresis could be minimized by selecting suitable hydrocolloids at optimized concentrations and incorporating appropriate additives to enhance water retention [34,44]. Syneresis of jelly products fortified with calcium L-threonate using a furcellaran–fish gelatin hydrogel system is presented in Table 2. It was noted that all jelly samples had no statistically significant differences (*p* > 0.05). These results indicated that neither furcellaran addition nor calcium L-threonate fortification measurably affected the percentage of expelled water. The stability observed in JFG-FUR and JFG-FUR-Ca indicated that furcellaran addition did not compromise network integrity; rather, the presence of sulfate groups within furcellaran likely enhanced electrostatic interactions with positively charged amino groups in gelatin, promoting tighter junction zones [45,46]. This interaction could restrict water mobility within the matrix, thereby preventing syneresis. Petcharat, Chaijan, Indriani, Pongsetkul, Karnjanapratum and Nalinanon [21] further reported that incorporating 25% furcellaran into a fish gelatin hydrogel could not negatively affect the syneresis of the resulting gel. Similarly, calcium L-threonate supplementation, either alone or combined with furcellaran, could not increase water release. Divalent calcium ions are known to form ionic bridges that strengthen polymer networks [43]. Therefore, the comparable syneresis observed in JFG-Ca and JFG-FUR-Ca might be explained by improved intermolecular structure (Figure 2), which contributed to water immobilization.

In addition, the consistently low syneresis observed across all jelly samples might also be attributed to the presence of sugar in the formulations. Sugar effectively suppresses water expulsion by lowering water activity, which reduces the amount of free, unbound water available for migration [47]. Moreover, sugar molecules form extensive hydrogen bonds with both water and biopolymer chains [48]. These interactions could enhance hydrogel cohesion and limit water mobility, thereby improving water-holding capacity. Therefore, the fish gelatin–furcellaran network was inherently stable against syneresis, and calcium fortification could not adversely alter water-holding capacity of jelly products. This was advantageous for product quality, as low syneresis was associated with desirable textural stability and prolonged shelf life during commercial storage and distribution of gel-based food systems.

### 2.4. Changes in the Appearance of Jelly Samples

**Figure 3:**
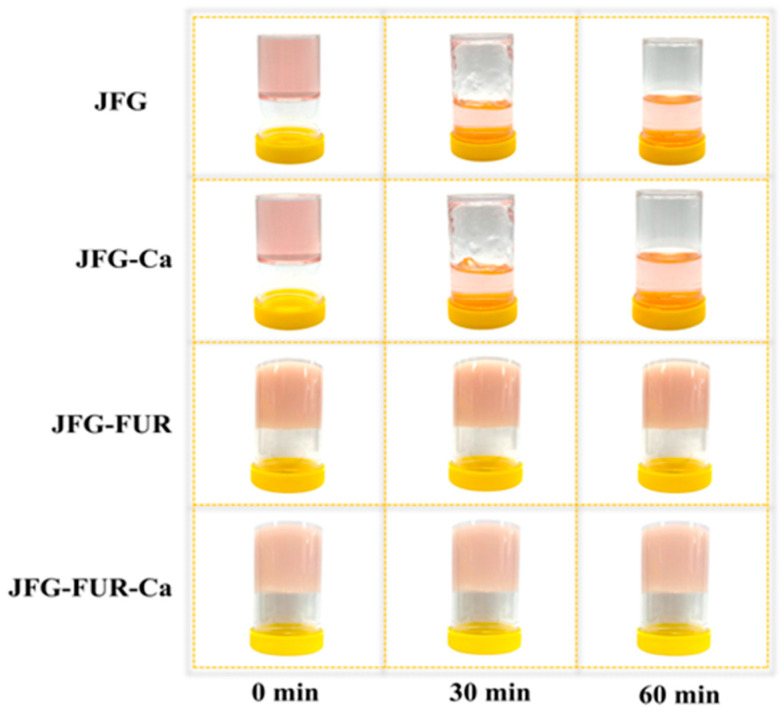
Appearance of jelly samples prepared using fish gelatin with and without other additives (furcellaran and calcium L-threonate) during storage at 30 °C for 60 min, illustrating gel stability. JFG = fish gelatin jelly, JFG-Ca = fish gelatin jelly with calcium L-threonate, JFG-FUR = fish gelatin jelly containing furcellaran, and JFG-FUR-Ca = fish gelatin–furcellaran jelly incorporated with calcium L-threonate.

The color attributes (L*, a*, b*, and ΔE*) of jelly products fortified with calcium L-threonate using a furcellaran–fish gelatin hydrogel system are shown in Table 3. According to the findings, no significant difference was observed between the JFG and JFG-Ca samples (*p* > 0.05). Both the JFG and JFG-Ca samples exhibited the highest L* value (*p* < 0.05). These results indicated that adding calcium L-threonate alone did not significantly alter product lightness. In contrast, the incorporation of furcellaran significantly decreased L* value in JFG-FUR, and the lowest L* value was found in the JFG-FUR-Ca sample (*p* < 0.05). This trend aligned with the duller and more opaque appearance of the resulting gels, as shown in Figure 3, suggesting that furcellaran integration increases gel turbidity through the formation of a denser network structure. Similar reductions in lightness (L*) after incorporation of polysaccharides in gelatin systems have been reported and are attributed to increased opacity and denser hydrogel networks [49,50].

The incorporation of furcellaran markedly influenced the chromatic properties of the jelly samples. Both a* and b* values significantly declined in formulations containing furcellaran, with the lowest redness and yellowness observed in JFG-FUR-Ca (*p* < 0.05). In contrast, JFG and JFG-Ca exhibited comparatively higher a* and b* values. The pronounced reduction in a* and b* suggested that diminished color saturation might be due to compact polysaccharide–gelatin network formation that restricted chromophore development and light absorption. Sanprasert, Kumnerdsiri, Seubsai, Lueangjaroenkit, Pongsetkul, Petcharat, Kaewprachu, Sai-ut, Rawdkuen, Teerapattarakan, Zhang, Jung and Kingwascharapong [34] noted that the intrinsic color of hydrocolloids, together with their dispersion behavior and intermolecular interactions, could strongly influence the visual characteristics of mixed gel systems. Correspondingly, ΔE* values (total color difference) increased in furcellaran-containing samples, reaching the highest in JFG-FUR-Ca, indicating substantial visual deviation from the control. From a consumer perception perspective, ΔE* values around 1.0 are generally considered minimally detectable, whereas ΔE* values of approximately 4.0 indicate clearly perceptible differences in color [51]. Therefore, these findings confirmed that furcellaran is the primary driver of chromatic modification in furcellaran–fish gelatin jelly products fortified with calcium L-threonate. In contrast, calcium L-threonate alone has only a minor effect on color attributes.

### 2.5. Rheological Behaviors of Jelly Samples

#### 2.5.1. Temperature Sweep

The gelling and melting temperatures of jelly samples prepared using fish gelatin with and without other additives (furcellaran and calcium L-threonate) are presented in Table 2. The JFG and JFG-Ca samples exhibited the lowest gelling temperatures among all formulations (*p* < 0.05), and neither was able to form a stable hydrogel under ambient conditions (25 °C). The result indicated that the gelatin–calcium L-threonate system alone could not provide sufficient intermolecular interactions to support gelation at room temperature, despite the increase in hydrogen and ionic bonding observed in the presence of calcium L-threonate (Figure 2). In contrast, the incorporation of furcellaran markedly increased the gelling temperature. JFG-FUR displayed a significantly higher gelling temperature, while the highest value was observed in JFG-FUR-Ca (*p* < 0.05). This demonstrated that the combined incorporation of furcellaran and calcium L-threonate synergistically enhanced network formation, thereby enabling rapid gel setting at room temperature. The improvement in gelling temperature is attributed to the formation of ionic and hydrogen interactions between gelatin and the sulfate groups present in furcellaran, a mechanism commonly reported in gelatin–polysaccharide hydrogels [10,34]. In addition, calcium ions may act as salt bridges within the mixed protein–polysaccharide network, contributing to the development of stronger junction zones and greater structural integrity [32]. A similar trend was observed for melting temperatures. JFG and JFG-Ca exhibited the lowest melting points, whereas JFG-FUR demonstrated a markedly higher value. The highest melting temperature was recorded for JFG-FUR-Ca (*p* < 0.05). These increases in gelling and melting temperatures were consistent with the intensified chemical interactions that supported the formation of a more robust three-dimensional gel network, as illustrated in Figure 2. Moreover, both JFG-FUR and JFG-FUR-Ca remained stable at temperatures above room temperature. Consistent with the appearance of jelly products fortified with calcium L-threonate using a furcellaran–fish gelatin hydrogel system (Figure 3), these samples could not melt during storage at 30 °C for 60 min. Notably, the incorporation of furcellaran and calcium L-threonate also maintained the thermoreversible characteristics typical of gelatin-based gels.

**Figure 4:**
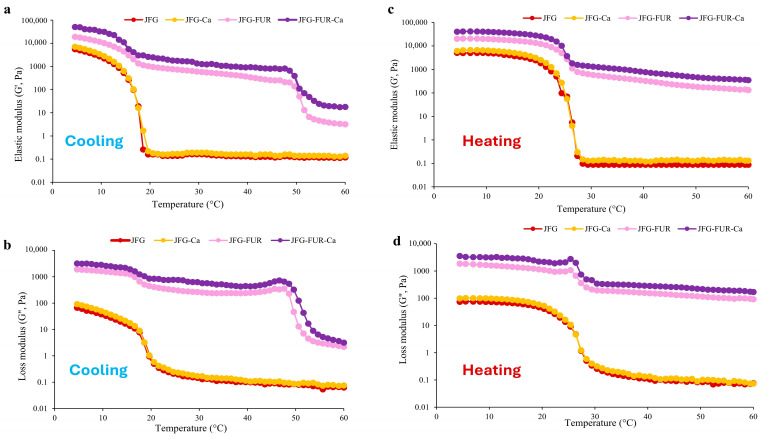
Elastic modulus (G′) (**a**) and loss modulus (G″) (**b**) of jelly samples during cooling from 60 to 5 °C. Elastic modulus (G′) (**c**) and loss modulus (G″) (**d**) jelly products fortified with calcium L-threonate using a furcellaran–fish gelatin hydrogel system during heating from 5 to 60 °C. JFG = fish gelatin jelly, JFG-Ca = fish gelatin jelly with calcium L-threonate, JFG-FUR = fish gelatin jelly containing furcellaran, and JFG-FUR-Ca = fish gelatin–furcellaran jelly incorporated with calcium L-threonate.

In addition, the rheological behavior of the jelly samples was evaluated through changes in the elastic modulus (G′) and loss modulus (G″) during cooling from 60 to 5 °C (Figure 4a,b) and subsequent heating from 5 to 60 °C (Figure 4c,d). All samples exhibited similar trends in G′ and G″ during both gelation and melting processes. Compared with the JFG sample, the jelly containing furcellaran showed higher values of both G′ and G″. Incorporation of furcellaran and calcium L-threonate further elevated these G′ and G″, which indicated an enhancement in the gel network structure [2]. Notably, the apparent change in the cooling profile between 60 and 50 °C for JFG-FUR and JFG-FUR-Ca corresponds to an early increase in both G′ and G″, indicating the onset of network development driven by furcellaran–gelatin intermolecular interactions. The presence of furcellaran promotes partial structuring at higher temperatures compared to JFG and JFG-Ca, while calcium further reinforces this network through ionic interactions, resulting in higher moduli throughout the cooling process. These results demonstrated that the combined addition of furcellaran and calcium L-threonate significantly improved the viscoelastic properties and thermal stability of the jelly products.

#### 2.5.2. Frequency Sweep

**Figure 5:**
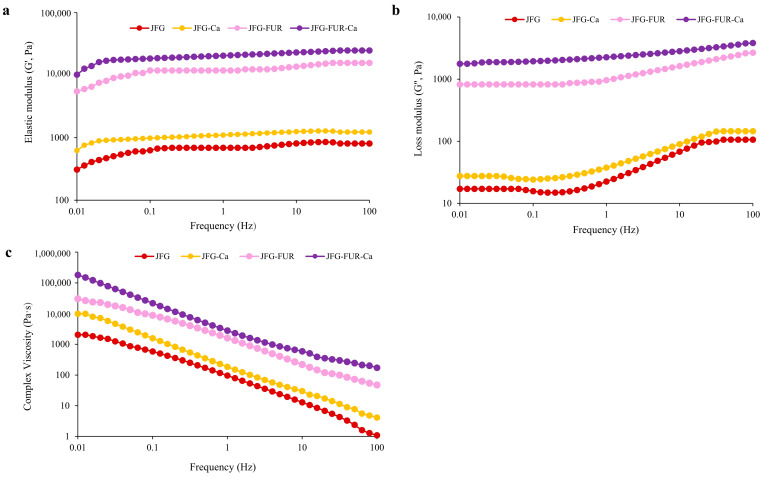
Elastic modulus (G′) (**a**) and loss modulus (G″) (**b**) and complex viscosity (**c**) of jelly samples as a function of frequency. JFG = fish gelatin jelly, JFG-Ca = fish gelatin jelly with calcium L-threonate, JFG-FUR = fish gelatin jelly containing furcellaran, and JFG-FUR-Ca = fish gelatin–furcellaran jelly incorporated with calcium L-threonate.

Figure 5 illustrates the frequency dependence of the elastic modulus (G′), loss modulus (G″), and complex viscosity (η*) of jelly products formulated with fish gelatin and furcellaran, with or without calcium L-threonate. In all samples, G′ values exceeded G″ across the frequency range. As reported by Kaur et al. [52], the materials exhibited a markedly higher storage modulus than loss modulus, which is indicative of solid-like behavior, characteristic of a proper gel system. The result also indicated the predominance of elastic behavior and confirmed the formation of a stable gel network [50]. The nearly constant elastic modulus (G′) observed across the frequency range indicated the absence of relaxation phenomena, suggesting that the intermolecular junctions within the functionalized hydrogel network were stable. This behavior implied that no significant structural rearrangement occurred, which would otherwise have led to variations in the G′ values [53]. The incorporation of furcellaran notably increased both G′ and G″ compared to the control (JFG). This referred to the enhancement of gel strength and structural rigidity due to the formation of intermolecular interactions between gelatin and furcellaran chains. The addition of calcium L-threonate further elevated these moduli, particularly in the JFG–FUR–Ca system. It confirmed that calcium ions acted as cross-linking agents that strengthened the polysaccharide–protein network [32]. The complex viscosity (η*) exhibited a decreasing trend with increasing frequency, consistent with shear-thinning (pseudoplastic) behavior of gels. The addition of furcellaran and calcium L-threonate increased the η* value of the resulting jelly. This trend also reconfirmed the presence of a stable hybrid hydrogel network [54]. Overall, these results demonstrated that the combined addition of furcellaran and calcium L-threonate improved the viscoelastic properties and network integrity of the gelatin-based jelly system.

### 2.6. Fourier Transform Infrared (FTIR) Spectra of Jelly Samples

**Figure 6:**
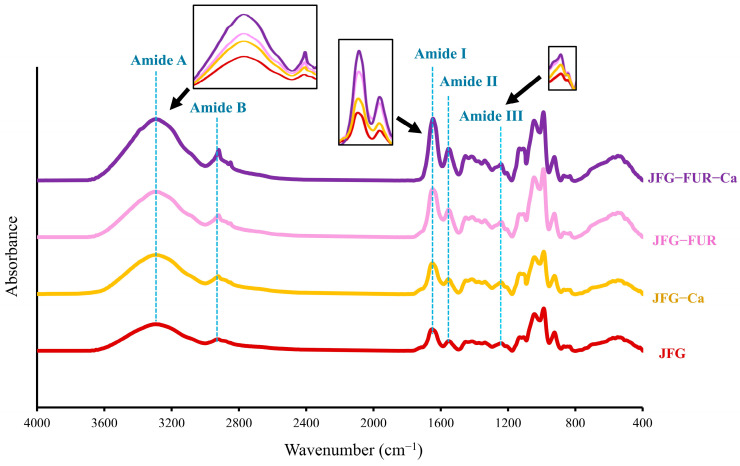
FTIR spectra of jelly samples. JFG = fish gelatin jelly, JFG-Ca = fish gelatin jelly with calcium L-threonate, JFG-FUR = fish gelatin jelly containing furcellaran, and JFG-FUR-Ca = fish gelatin–furcellaran jelly incorporated with calcium L-threonate.

Generally, FTIR provides qualitative and indirect insight into molecular interactions via functional group vibrations, rather than direct quantitative determination of specific bonds or protein secondary structures [55]. The FTIR spectra of jelly products formulated using the furcellaran–fish gelatin hydrogel system with and without calcium L-threonate are presented in Figure 6. All samples exhibited the characteristic absorption bands associated with gelatin. The amide I, II, and III bands appeared at approximately 1646, 1554, and 1240 cm^−1^, corresponding to C=O stretching, N–H bending coupled with C–N stretching, and a combination of C–N stretching and N–H deformation, respectively [32,50]. The amide A band was observed near 3290–3300 cm^−1^ due to N–H stretching linked to hydrogen bonding, while the amide B band at 2928 cm^−1^ was associated with CH_2_ stretching vibrations [32]. Distinct absorption bands at approximately 1250, 924, and 847 cm^−1^ emerged in furcellaran-containing samples. These peaks could confirm the incorporation of sulfate ester groups, 3,6-anhydrogalactose, and D-galactose residues characteristic of furcellaran [46,50]. It has also verified the successful integration of furcellaran into the protein–polysaccharide network. In addition to furcellaran-derived peaks, all jelly formulations exhibited a distinct band at 1030–1100 cm^−1^, attributable to C–O stretching vibrations of carbohydrates such as glucose and fructose [56], which are naturally present as sweetening agents in the jelly products. The persistence of this peak across formulations indicates that carbohydrate constituents contributed to the overall vibrational fingerprint of the product.

Co-addition of furcellaran and calcium L-threonate led to pronounced changes in the amide A and B regions. The amide A band shifted to slightly higher wavenumbers, with a concurrent increase in intensity in the JFG-FUR-Ca sample. This might be caused by the enhanced hydrogen bonding between gelatin and polysaccharide chains [57], which was further strengthened by Ca^2+^ coordination. These findings aligned with the hydrogen-bonding analysis (Figure 2), which showed increased hydrogen-bonding interactions as furcellaran and calcium ion were incorporated into the jelly sample. The amide B band also shifted to a lower wavenumber (from 2928 to 2921 cm^−1^) and showed the highest intensity in the JFG-FUR-Ca sample. This change suggested a stronger involvement of CH_2_-containing side groups in intermolecular associations [50]. It was noted that incorporating furcellaran and calcium L-threonate into the jelly sample resulted in higher intensities of the amide I, II, and III bands, which reflected improved molecular interactions within the polymer structure. The amide I region also shifted upward, moving from 1646 cm^−1^ in the JFG sample to 1647, 1649, and 1651 cm^−1^ in the JFG-Ca, JFG-FUR, and JFG-FUR-Ca samples, respectively. Derkach et al. [58] suggested that the elevation of the amide I band demonstrated a reduction in the ordered secondary structure of gelatin, presumably resulting from complex formation [58]. This alteration could be elucidated by the electrostatic attraction between the sulfate groups in furcellaran and the positively charged amide groups in fish gelatin, which facilitated partial structural rearrangement and resulted in a more interconnected heterogeneous network [59]. In addition, Ca^2+^ further reinforced these interactions by acting synergistically with furcellaran and fish gelatin, thereby enhancing the overall structural stability of the resulting jelly. The structural implications observed in the FTIR spectra correspond well with the greater gel strength and hardness observed in JFG-FUR-Ca (Figure 1 and Table 1). These findings reconfirmed that the structural reinforcement derived from polysaccharide–protein–ion interactions. Moreover, a strong, broad O–H stretching band around ~3255 cm^−1^ confirmed extensive hydrogen bonding within the calcium-fortified jelly [46]. Overall, the FTIR findings demonstrated that the combined effects of furcellaran and calcium L-threonate promoted strong molecular interactions, leading to enhanced structural integrity and improved gelling properties of the calcium-fortified jelly matrix.

### 2.7. Microstructure of Jelly Samples

**Figure 7:**
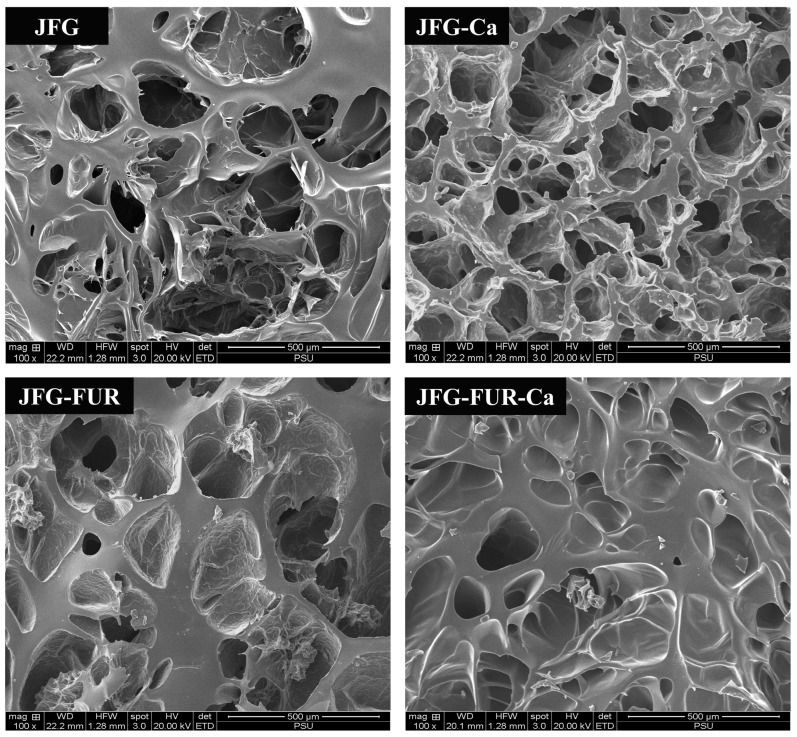
Microstructures of jelly samples. Magnification: 100×. JFG = fish gelatin jelly, JFG-Ca = fish gelatin jelly with calcium L-threonate, JFG-FUR = fish gelatin jelly containing furcellaran, and JFG-FUR-Ca = fish gelatin–furcellaran jelly incorporated with calcium L-threonate.

The SEM micrographs of jelly products prepared using different hydrogel formulations revealed distinct microstructural features, as shown in Figure 7. In general, the spatial configuration of gelatin molecules and the hydroxyproline content within the matrix are known to influence gel rigidity [60]. Previous studies have demonstrated that gelatin with a loosely packed and coarse network generally exhibited lower bloom strength [61]. Consistent with this, the SEM micrographs of the fish gelatin control (JFG) displayed an irregular, highly porous structure with large and discontinuous voids (Figure 7). This disorganized structure was indicative of limited intermolecular interactions, which might contribute to the lower gel strength commonly observed in fish gelatin–based gels. Incorporation of calcium L-threonate alone (JFG-Ca) produced noticeable but limited improvement in matrix organization. The porous network remained heterogeneous; however, the pore edges appeared slightly more compact. The result suggested that Ca^2+^ ions participated in weak electrostatic interactions with gelatin chains. These interactions likely modestly reinforced network crosslinking but were insufficient to achieve substantial microstructural consolidation. It was noted that the sample containing furcellaran (JFG-FUR) exhibited a more compact and uniform structure. The internal network appeared denser, with smaller and more interconnected pores. Moreover, the microstructure of JFG-FUR-Ca demonstrated the highest degree of compactness. The gel matrix displayed fewer voids and smoother, continuous surfaces, indicating extensive crosslinking within the polymer network. This improved organization indicated strong intermolecular associations between furcellaran and gelatin, mainly through hydrogen bonding and ionic interactions, as evidenced in Figure 2. Dong et al. [62] supported that the reduction in pore size was due to crosslinking among gelatin chains, which strengthens intermolecular interactions and results in a more compact internal hydrogel structure [62]. In addition, Petcharat et al. [63] reported that furcellaran could also function as a structural filler within the three-dimensional network of protein. This function could promote the formation of a denser and more compact gel matrix in the fish gelatin-furcellaran composite system [21]. The combination of calcium L-threonate into fish gelatin-furcellaran hydrogel facilitated ionic interactions with carboxyl groups in furcellaran and polar amino residues in gelatin, resulting in a more integrated three-dimensional network. The improved structural uniformity was correlated well with the increased gel strength and hardness observed in JFG-FUR-Ca (Figure 1 and Table 1). Thus, the SEM findings reconfirmed the synergistic effect of furcellaran–gelatin interactions reinforced by Ca^2+^-mediated crosslinking in jelly products.

### 2.8. Sensory Evaluation of Jelly Samples

The sensory likeness scores of jelly products fortified with calcium L-threonate using a furcellaran–fish gelatin hydrogel system are summarized in Table 4. The control (JFG) and calcium-fortified sample (JFG-Ca) received the highest ratings for all attributes, except for odor acceptability (*p* < 0.05). These results indicated that calcium L-threonate addition alone had not adversely affected the sensory quality of the fish gelatin jelly. This finding agrees with previous results, in which JFG-Ca exhibited gel strength and texture profile analysis comparable to those of the control. This implied that ionic crosslinking by calcium ions reinforced the gelatin network without impairing elasticity or transparency. In contrast, the addition of furcellaran (JFG-FUR and JFG-FUR-Ca) significantly reduced likeness scores for appearance and color compared with the control (JFG) (*p* < 0.05). The reduced appearance and color likeness scores corresponded to the decreased L* value and increased opacity observed in Table 3 and Figure 3. The results suggested that furcellaran incorporation altered the optical properties of the jelly product. It was observed that the incorporation of furcellaran, either alone or in combination with calcium L-threonate, increased the odor likeness scores while decreasing the taste likeness scores compared with the control (*p* < 0.05). The improvement in odor perception might be attributed to a dilution effect, whereby furcellaran reduces the characteristic fishy odor of fish gelatin [64]. Conversely, the lower taste scores might be related to altered flavor release and the thicker oral texture induced by furcellaran, which could influences sweetness perception and the diffusion of volatile compounds [65].

Moreover, the furcellaran-containing samples showed lower firmness, springiness, and mouthfeel likeness scores. This trend might be attributed to the incorporation of furcellaran, which generated a more rigid and compact gel matrix [21], especially when combined with calcium ion. Both JFG-FUR and JFG-FUR-Ca displayed a denser and less porous microstructure (Figure 7), accompanied by strengthened intermolecular interactions (Figure 2 and Figure 6) and a reduced ability to undergo elastic deformation (Table 1). The decrease in mouthfeel likeness might also be related with higher melting temperatures in the JFG-FUR and JFG-FUR-Ca samples. Both formulations exhibited melting temperatures above 37 °C, which prevented them from melting readily in the oral cavity. Godoi et al. [66] reported that the melt-in-mouth characteristic of gelatin resulted from the disruption of hydrogen bonds within its gel network when exposed to body temperature. The loss of this property is considered one of the most desirable sensory attributes of gelatin gels and contributed to the lower mouthfeel acceptability observed in the furcellaran-containing samples. Notably, the higher melting temperatures and reduced melt-in-mouth perception of the furcellaran-containing formulations might be advantageous for non-melting jelly products intended for warm climates or ambient-temperature storage, in which improved thermal stability is preferred over rapid oral melting. Overall acceptability was slightly lower in the furcellaran-containing samples, with JFG-FUR-Ca showing the lowest score (*p* < 0.05). Nevertheless, the JFG-FUR-Ca sample still achieved overall likeness scores above 7.0 (liked moderately). Thus, although the addition of furcellaran and calcium L-threonate influenced certain sensory characteristics, the overall consumer response remained positive, supporting the suitability of these formulations for jelly applications.

### 2.9. Calcium Bioavailability of Jelly Samples After Digestion Through Gastrointestinal Tract Model System

**Figure 8:**
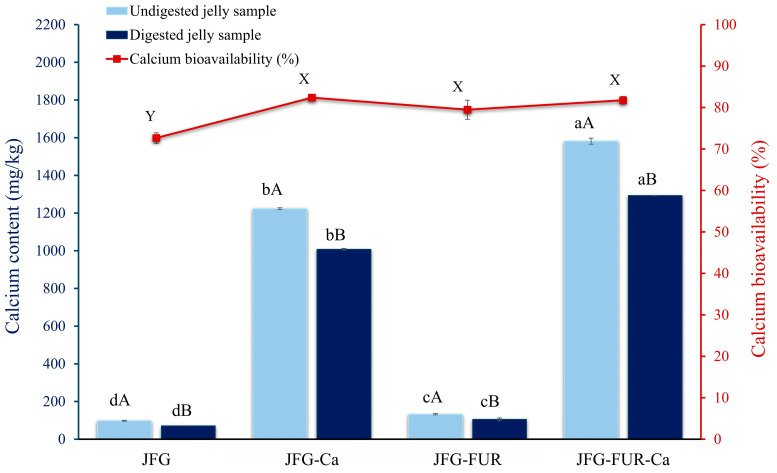
Calcium content of undigested and digested jelly samples through the gastrointestinal tract model. Bars represent the standard deviation (*n* = 3). A, B on the bars indicate significant differences (*p* < 0.05) between undigested and digested jelly samples within the same condition (intragroup comparison), while a–d indicate significant differences (*p* < 0.05) of the same digestion condition across different jelly formulations (intergroup comparison). The line represents calcium bioavailability (%) of jelly samples. X, Y on the line indicates significant differences (*p* < 0.05). JFG = fish gelatin jelly, JFG-Ca = fish gelatin jelly with calcium L-threonate, JFG-FUR = fish gelatin jelly containing furcellaran, and JFG-FUR-Ca = fish gelatin–furcellaran jelly incorporated with calcium L-threonate.

The calcium contents of the jelly samples before and after digestion through the gastrointestinal tract model system are presented in Figure 8. Before digestion, a distinct difference was evident between jellies without added calcium L-threonate (JFG and JFG-FUR) and those fortified with the calcium L-threonate (JFG-Ca and JFG-FUR-Ca). The fortified samples exhibited significantly higher calcium contents (*p* < 0.05) than their non-fortified counterparts, indicating effective incorporation of calcium L-threonate into the jelly matrix. The small amount of calcium observed in the non-fortified formulations is attributable to the trace amounts of naturally occurring calcium inherently present in fish gelatin and furcellaran [67,68]. It was highlighted that the JFG-FUR-Ca sample provided 158.15 mg of calcium per 100 g, equivalent to 15.81% of the recommended daily intake (RDI). According to the Thai Ministry of Public Health, products delivering more than 15% of the 1000 mg calcium RDI could be classified as a “*good source of calcium*”. Thus, the JFG-FUR-Ca formulation met this criterion and represented a promising calcium-fortified functional jelly [69].

Across all sample types, digestion significantly reduced the calcium content (*p* < 0.05). The observed reduction in calcium content of digested jelly samples might be attributed to the propensity of calcium ions to interact with components present in simulated gastrointestinal fluids, resulting in the formation of insoluble precipitates during enzymatic hydrolysis [70]. Typically, fortified calcium forms become less soluble as they transition from the highly acidic stomach to the neutral intestinal environment, which is critical for absorption [71]. Despite this reduction, the fortified jellies, especially JFG-FUR-Ca, retained substantially higher calcium levels than non-fortified samples following digestion (*p* < 0.05). This result showed the strong calcium-holding capacity of hydrogels containing both fish gelatin and furcellaran, even in the presence of digestive enzymes. Calcium bioavailability, calculated as the ratio of calcium retained after digestion to the amount initially present, remained high (approximately 70–80%) among all fortified formulations. Notably, no significant differences were observed between JFG-Ca and JFG-FUR-Ca (*p* > 0.05). The results indicated that the enhanced gel network formed by increased formulation complexity with furcellaran did not impair calcium absorption efficiency. The high bioavailability achieved here exceeded the values reported for several plant-derived calcium fortificants, including okra extract, which previously demonstrated bioavailability below 60% [72]. In conclusion, these findings supported the suitability of the fortified fish gelatin–furcellaran jelly as a promising functional product for calcium supplementation, particularly for populations with increased dietary calcium requirements.

## 3. Conclusions

The incorporation of furcellaran and calcium L-threonate into fish gelatin-based jelly effectively enhanced gel strength, hardness, thermal stability, and calcium bioavailability, while maintaining low syneresis and acceptable sensory properties. The synergistic interactions between gelatin, furcellaran, and calcium ions reinforced the gel network through ionic and hydrogen bonding, enabling stable room-temperature gelation and improved structural integrity. These findings demonstrated that this formulation strategy provided a functional and nutritionally fortified jelly suitable for calcium supplementation. Moreover, this approach presents a sustainable and innovative avenue for the development of fortified foods, contributing to nutritional security, public health, and the promotion of sustainable food systems in line with the Sustainable Development Goals (SDGs). Future studies should focus on evaluating shelf-life stability under commercial storage conditions, optimizing flavor and sweetness perception, and assessing consumer acceptance to facilitate industrial application.

## 4. Materials and Methods

### 4.1. Materials

Fish gelatin, derived from tilapia skin, had about 240 g bloom strength and originated from Lapi Gelatine S.p.A. (Lapi Gelatine S.p.A., Empoli, Italy). Furcellaran Estgel 1000 (food grade) was obtained from Est-Agar AS, situated in Karla village, Estonia. The molecular weight was 2.55 × 10^5^ Da, and 9.7% moisture was utilized without further purification. At a concentration of 2.5%, furcellaran exhibited a bloom strength of 480 g. Calcium L-threonate, exhibiting a purity of 99.8%, comprised a total calcium (as Ca) content of 12.92% by weight and was acquired from Power Tech Chemical Industry Co., Ltd. (Bangkok, Thailand). The sucrose, citric acid and lychee flavor powder were of food analysis grade.

### 4.2. Preparation of the Jelly Samples

Jelly products were prepared using the basic formulation: fish gelatin (10 g), sucrose (20 g), water (100 mL), citric acid (1 g), and lychee flavor powder (1 mg). Variations were produced by partially substituting fish gelatin with furcellaran (2.5 g) and by incorporating calcium L-threonate (1200 mg). To prepare the jelly samples, furcellaran and fish gelatin powders were separately dispersed in deionized water and then heated to 90 °C and 60 °C, respectively. The two solutions were combined and stirred for 10 min until homogeneous. Calcium L-threonate, sucrose, lychee flavor powder and citric acid were subsequently added to the mixture, which was stirred using a magnetic stirrer (Heidolph Instruments GmbH & Co. KG, Schwabach, Germany) until complete homogeneity was obtained. The resulting mixtures were maintained at 60 °C before further analyses. The prepared jelly samples were designated as JFG (fish gelatin jelly), JFG-Ca (fish gelatin jelly with calcium L-threonate), JFG-FUR (fish gelatin jelly containing furcellaran), and JFG-FUR-Ca (fish gelatin–furcellaran jelly with calcium L-threonate). For gel preparation, the mixtures were poured into cylindrical molds measuring 3 cm in diameter and 2.5 cm in height. The molded samples were then stored at 4 °C for 18 h to allow proper setting before analyses. The pH of the jelly samples was subsequently measured and recorded using a pH meter (Metrohm, Herisau, Switzerland).

### 4.3. Analyses

#### 4.3.1. Texture Measurement

Texture properties of the jelly sample were evaluated using a texture analyzer (TA-XT Plus, Stable Micro Systems, Surrey, UK). Gel strength was determined with a 5 kg load cell using a 1.27 cm flat-faced cylindrical Teflon^®^ plunger, and the maximum force (g) required to penetrate 4 mm into the gel at 8–10 °C was recorded [17]. Texture profile analysis (TPA) was performed using a P/50 probe with a 50 kg load cell, applying two compression cycles to 50% of the original height at a crosshead speed of 0.5 mm/s, with a 10 s interval between cycles. Textural parameters were obtained from the force–time curves at 8–10 °C [34].

#### 4.3.2. Evaluation of Intermolecular Forces

All jelly samples were subjected to examine the intermolecular forces as the method suggested by Wang et al. [73]. One gram of jelly sample was homogenized in 10 mL of SSA (0.05 mol/L NaCl), SSB (0.6 mol/L NaCl), SSC (0.6 mol/L NaCl + 1.5 mol/L urea), and SSD (0.6 mol/L NaCl + 8 mol/L urea). The mixtures were then centrifuged at 1600 rpm for 4 min at 4 °C using a temperature-controlled centrifuge (Eppendorf 5920R, Eppendorf North America Inc., Hamburg, Germany), and the supernatants were collected [73,74]. Protein concentrations were quantified using the Folin reagent. Differences between SSB–SSA, SSC–SSB, and SSD–SSC represented ionic bonding, hydrogen bonding, and hydrophobic interactions, respectively.

#### 4.3.3. Rheological Analysis

The rheological properties were conducted in accordance with the procedure suggested by Huang, Tu, Sha, Wang, Hu and Hu [32] by using a rheometer (Physica MCR301, Anton Paar GmbH, Graz, Austria) fitted with a cup and bob (CC27 geometry). The diameter of the cup was 28.92 mm, and the diameter of the bob was 26.56 mm. A jelly mixture (17 mL) was transferred into a cup and bob, and frequency sweep tests were carried out at 25 °C and 1% strain over 0.01–100 Hz in a defined linear viscoelastic region. The temperature sweep analyses were conducted from 60 to 5 °C and 5 to 60 °C with a temperature ramp of 1 °C/min. The measurements were carried out at 1% strain and 1 Hz frequency. The elastic modulus G′ and the loss (viscous) modulus G″ were recorded. Finally, the gelling and melting temperatures were determined as the temperatures at which tan δ (=G″/G′) became 1 (or δ = 45°).

#### 4.3.4. Determination of Color

The Ultrascan XE, Hunter lab colorimeter (Hunter Lab Inc., Reston, VA, USA) was employed to determine the color of jelly. The L*, a*, and b* of jelly were defined. The total difference in color (∆E*) was evaluated using the method of Rawdkuen et al. [75].

#### 4.3.5. Measurement of Syneresis

The syneresis of all jelly samples was evaluated as recommended by Sanprasert et al. [76]. The mass of the graduated centrifuge tubes absence of a sample (S1), the mass of the gels along with the tubes post-centrifugation (S2), and the initial mass of the sample (S3) were taken into account. The syneresis of the hydrogel was calculated using the formula (S1 − S2)/S3 and expressed as a percentage.

#### 4.3.6. FTIR Spectroscopy

The FTIR spectra of the dehydrated samples were measured following the method of Indriani et al. [77] using an Invenio-s attenuated total reflectance (ATR) FTIR spectrometer (Bruker Co., Ettlingen, Germany) equipped with a Bruker A225/Q Platinum ATR unit (Bruker Optik GmbH, Rosenheim, Germany) and multiple ATR crystal diamond cells. Via OPUS 8.5 (Bruker Co., Ettlingen, Germany), the spectrum range of 4000–400 cm^−1^ was investigated at a resolution of 4 cm^−1^ [78].

#### 4.3.7. Scanning Electron Microscopy (SEM)

Scanning electron microscopy (Quanta 400; FEI, Eindhoven, The Netherlands) was used to observe the microstructure of the jelly sample, following the procedure described by Somjid et al. [79]. Dried specimens were mounted on bronze stubs, sputter-coated with gold, and examined under an accelerating voltage of 20 kV.

#### 4.3.8. Sensory Evaluation

Sensory evaluation was carried out with approval from the Research Ethics Committee of King Mongkut’s Institute of Technology, Ladkrabang, Bangkok, Thailand (Protocol No. EC-KMITL_67_109). Fifty untrained panelists familiar with jelly products participated in the study. Each sample (1.0 cm thick, 2.5 cm in diameter) was served in a covered white plastic cup, labeled with a three-digit random code, and stored at 8–10 °C until assessment. Panelists rated appearance, color, odor, firmness, springiness, taste, mouth feel and overall liking using a 9-point hedonic scale [80]. Drinking water at room temperature was provided for palate cleansing between samples.

#### 4.3.9. Influence of Gastrointestinal Tract Model System on the Calcium Bioavailability of Jelly Products

An in vitro gastrointestinal tract model was established following Petsong, Yarnpakdee, Senphan, Sriket, Kingwascharapong, Moula Ali, Surya and Karnjanapratum [71] with slight adjustments. The samples were mixed with 100 mL of distilled water and 0.5 mL of 1 M HCl–KCl buffer (pH 1.5), then 5 mL of pepsin solution (32 U/mL in 1 M HCl–KCl buffer, pH 1.5) was added. The mixture was incubated at 37 °C for 2 h with continuous agitation to simulate stomach digestion. Subsequently, the pH was adjusted to 6.8 using 1 M NaHCO_3_ (1 mL), and an enzyme mixture containing pancreatin (10 mg/mL), trypsin (14,600 U/mL), and bile extract (13.5 mg/mL) in 10 mM phosphate buffer (pH 8.2) (1 mL) was added to mimic intestinal digestion. The reaction was then incubated at 37 °C for 3 h under constant shaking. Digestion was terminated by heating in a boiling water bath for 10 min, after which samples were stored at 4 °C. Calcium content in digested and undigested samples was determined according to the procedure of Benjakul et al. [81].

### 4.4. Statistical Analysis

All experiments were conducted in triplicate following a completely randomized design (CRD), except for the sensory evaluation, which applied a randomized complete block design (RCBD). Differences among sample means were assessed using Duncan’s multiple range test. Statistical analyses were carried out using SPSS Statistics Software Version 28 (SPSS Inc., Chicago, IL, USA), with significance defined at *p* < 0.05.

## Figures and Tables

**Table 1:** Texture profile analysis (TPA) of jelly samples prepared using fish gelatin with and without other additives (furcellaran and calcium L-threonate).

Samples	Hardness (N)	Springiness	Cohesiveness	Gumminess (N)	Chewiness (N × mm)
JFG	26.21 ± 0.70 ^d^	0.93 ± 0.02 ^a^	0.87 ± 0.04 ^a^	22.81 ± 1.46 ^d^	21.29 ± 1.55 ^d^
JFG-Ca	30.55 ± 0.68 ^c^	0.93 ± 0.01 ^a^	0.86 ± 0.03 ^a^	26.12 ± 0.90 ^c^	24.19 ± 0.86 ^c^
JFG-FUR	66.41 ± 1.31 ^b^	0.89 ± 0.01 ^b^	0.77 ± 0.01 ^b^	51.33 ± 0.84 ^b^	45.59 ± 1.16 ^b^
JFG-FUR-Ca	78.14 ± 1.76 ^a^	0.86 ± 0.01 ^c^	0.71 ± 0.01 ^c^	55.55 ± 2.19 ^a^	47.89 ± 2.51 ^a^

Different lowercase superscript letters in the column indicate significant differences (*p* < 0.05). Values are presented as mean ± SD (*n* = 10). JFG = fish gelatin jelly, JFG-Ca = fish gelatin jelly with calcium L-threonate, JFG-FUR = fish gelatin jelly containing furcellaran, and JFG-FUR-Ca = fish gelatin–furcellaran jelly incorporated with calcium L-threonate.

**Table 2:** Syneresis, gelling, and melting temperatures of jelly samples prepared using fish gelatin with and without other additives (furcellaran and calcium L-threonate).

Samples	Syneresis (%)	Gelling Temperature (°C)	Melting Temperature (°C)
JFG	0.04 ± 0.00 ^a^	19.69 ± 0.29 ^c^	26.28 ± 0.28 ^c^
JFG-Ca	0.04 ± 0.01 ^a^	20.03 ± 0.38 ^c^	27.06 ± 0.60 ^c^
JFG-FUR	0.04 ± 0.00 ^a^	41.27 ± 0.57 ^b^	44.68 ± 0.51 ^b^
JFG-FUR-Ca	0.04 ± 0.00 ^a^	44.94 ± 0.58 ^a^	50.23 ± 0.39 ^a^

Different lowercase superscript letters in the column indicate significant differences (*p* < 0.05). Values are presented as mean ± SD (*n* = 3). JFG = fish gelatin jelly, JFG-Ca = fish gelatin jelly with calcium L-threonate, JFG-FUR = fish gelatin jelly containing furcellaran, and JFG-FUR-Ca = fish gelatin–furcellaran jelly incorporated with calcium L-threonate.

**Table 3:** Color values of jelly samples prepared using fish gelatin with and without other additives (furcellaran and calcium L-threonate).

Samples	L*	a*	b*	∆E*
JFG	63.85 ± 0.47 ^a^	14.49 ± 0.34 ^a^	9.49 ± 0.23 ^a^	34.68 ± 0.54 ^c^
JFG-Ca	63.28 ± 0.94 ^a^	13.97 ± 0.36 ^a^	9.22 ± 0.42 ^a^	35.39 ± 0.64 ^c^
JFG-FUR	49.50 ± 0.19 ^b^	3.29 ± 0.08 ^b^	2.86 ± 0.12 ^b^	44.65 ± 0.15 ^b^
JFG-FUR-Ca	43.88 ± 0.72 ^c^	2.57 ± 0.13 ^c^	1.07 ± 0.03 ^c^	50.20 ± 0.70 ^a^

Different lowercase superscript letters in the column indicate significant differences (*p* < 0.05). Values are presented as mean ± SD (*n* = 10). JFG = fish gelatin jelly, JFG-Ca = fish gelatin jelly with calcium L-threonate, JFG-FUR = fish gelatin jelly containing furcellaran, and JFG-FUR-Ca = fish gelatin–furcellaran jelly incorporated with calcium L-threonate.

**Table 4:** Likeness score of jelly samples prepared using fish gelatin with and without other additives (furcellaran and calcium L-threonate).

Sample	Appearance	Color	Odor	Firmness	Springiness	Taste	Mouth Feel	Overall
JFG	8.12 ± 0.85 ^a^	8.22 ± 1.30 ^a^	6.52 ± 1.82 ^b^	8.26 ± 1.60 ^a^	8.00 ± 1.48 ^a^	7.30 ± 1.45 ^a^	7.86 ± 1.83 ^a^	8.42 ± 1.23 ^a^
JFG-Ca	8.26 ± 0.92 ^a^	8.28 ± 1.28 ^a^	6.60 ± 1.53 ^b^	8.04 ±1.46 ^a^	8.02 ± 1.24 ^a^	7.02 ± 2.32 ^ab^	7.54 ± 1.40 ^a^	8.28 ± 1.18 ^a^
JFG-FUR	7.60 ± 1.14 ^b^	7.68 ± 1.04 ^b^	7.76 ± 1.29 ^a^	7.34 ±1.22 ^b^	7.18 ± 1.04 ^b^	6.42 ± 1.67 ^b^	5.76 ± 2.02 ^b^	7.66 ± 1.02 ^b^
JFG-FUR-Ca	7.70 ± 1.16 ^b^	7.82 ± 1.22 ^b^	7.36 ± 1.59 ^a^	7.28 ± 1.08 ^c^	6.62 ± 0.75 ^c^	6.46 ± 1.42 ^b^	6.52 ± 1.61 ^c^	7.52 ± 1.09 ^b^

Different lowercase superscript letters in the column indicate significant differences (*p* < 0.05). Values are presented as mean ± SD. JFG = fish gelatin jelly, JFG-Ca = fish gelatin jelly with calcium L-threonate, JFG-FUR = fish gelatin jelly containing furcellaran, and JFG-FUR-Ca = fish gelatin–furcellaran jelly incorporated with calcium L-threonate.

## Data Availability

The data presented in this study are available in this article. Further inquiries can be directed to the corresponding author.
